# Intraoperative periprosthetic femoral fracture in cementless hip hemiarthroplasty for femoral neck fracture does not change long-term outcomes

**DOI:** 10.1051/sicotj/2025045

**Published:** 2025-08-07

**Authors:** Nissan Amzallag, Itay Ashkenazi, Mohamed Abadi, Nadav Graif, Yaniv Warschawski

**Affiliations:** Division of Orthopedics, Tel Aviv Sourasky Medical Center, Tel-Aviv, affiliated to the Sackler Faculty of Medicine Tel Aviv University 6 Weitzman St. Tel Aviv 6423906 Israel

**Keywords:** Hip hemiarthroplasty, Intraoperative periprosthetic fracture

## Abstract

*Purpose*: Intraoperative periprosthetic femoral fracture (IPFF) is a known complication during hemiarthroplasty (HA), which may lead to inferior outcomes. Few studies have assessed the outcomes of IPFF in HA for displaced femoral neck fractures (FNF). This study aims to evaluate the incidence of IPFF in cementless HA for displaced FNF and compare long-term outcomes between patients with and without IPFF. *Methods*: We retrospectively reviewed institutional surgical data of patients who underwent cementless HA for displaced FNF from January 2010 to January 2022. The presence, location, and treatment of IPFF, as well as the effect of IPFF on postoperative weight-bearing, status were assessed. Mortality, readmission, and revision rates were compared between the IPFF and non-IPFF group. *Results*: A total of 1,586 patients were included in the study. 104 patients (6.6%) in the IPFF group vs. 1,482 patients (93.4%) in the non-IPFF group. The IPFF location was mostly the calcar (59.6%), followed by the greater trochanter (35.5%) and the femoral shaft (8.6%). Most fractures were treated with fixation (92.3%) and full weight-bearing postoperatively (95.1%). Surgery duration was longer in the IPFF group (*p* < 0.001). However, there were no significant differences between groups regarding 30-day, 90-day, and 1-year mortality rates, 90-day readmission rates, or revision rates at the latest follow-up. A multivariate binary logistic regression found similar long-term results. *Conclusions*: While IPFF remains a recognized complication of cementless HA for displaced FNF, its occurrence does not adversely affect long-term outcomes when appropriately managed.

## Introduction

Femoral neck fractures (FNF) in the elderly represent a global public health concern with an increasing incidence and associated morbidity and mortality. The standard of care for displaced FNF includes hemiarthroplasty (HA) or total hip arthroplasty (THA). The choice between HA and THA depends on several factors, including patient age, functional level, cognitive status, pre-existing arthritis, and comorbidities [1–4].

Intraoperative periprosthetic femoral fracture (IPFF) is a well-described complication of THA, with its incidence varying widely. It has been reported to be as low as 0.1–1% for cemented THA and nearly 2.95% for cementless primary THA, reaching up to 27.8% in high-risk patients [5–7]. This variation in prevalence may be attributed to differences in demographic factors, the wide range of femoral implant types, patients’ surgical histories, and the varying levels of surgeon expertise [8, 9].

Although most IPFFs are simple, non-displaced fractures [10], an unidentified IPFF may lead to inferior outcomes, including early loosening, necessitating further revision surgery [5, 11]. Previous studies have identified risk factors for IPFF in THA patients, including age [9, 12, 13], female sex [9, 12–14], osteoporosis [12, 15] and rheumatoid arthritis [15]. Nonetheless, the use of cemented [9, 14–17] collared [18] femoral stems and conventional over compaction broaching [19] has previously been shown to decrease the incidence of IPFF.

To date, only a few studies have assessed IPFF in HA for displaced FNF [20–23], and information on its incidence and risk factors are rarely described [16, 24, 25]. To our knowledge, no studies have compared the long-term outcomes for these patients. The aim of this study is to assess the risk of IPFF in cementless HA for displaced FNF and to compare short- and long-term outcomes between patients who had IPFF and those who did not. We hypothesize that IPFF after cementless HA for displaced FNF does not result in significant long-term clinical differences.

## Methods

### Study design

The present study was approved by the institutional review board (IRB no. 0566-23-TLV). It is a retrospective study based on data from an institutional registry at a high-volume, tertiary academic center, covering the period from January 2010 to January 2022. The inclusion criteria for this study were: (1) patients older than 65 years, (2) Patients who underwent cementless HA for displaced FNF. Exclusion criteria were: (1) patients with less than one year of follow-up for other reasons than mortality, (2) patients with previous surgery on the operated hip, (3) patients with a pathological fracture.

### Data collection

Data were collected through a review of electronic medical records, including surgical notes, admission and discharge summaries, hospitalization and outpatient follow-up, readmission data, and revision surgeries (e.g., due to prosthetic joint infection (PJI), dislocation, aseptic loosening, or periprosthetic fractures). Baseline patient characteristics, including age, sex, body mass index (BMI), American Society of Anaesthesiology (ASA) score, and Charlson comorbidity index (CCI), were retrieved from the institutional database. Proximal femoral bone quality was assessed using the Dorr classification. Mortality data were obtained from the hospital database, linked with the national mortality registry. IPFF data were manually extracted from patients’ medical records and included fracture presence, classification, treatment, postoperative rehabilitation protocols, and weight-bearing status. Surgical time was recorded from skin incision to wound closure. Final follow-up was defined as the most recent orthopaedic evaluation. All data were securely stored in an encrypted Excel file (Microsoft, Redmond, WA). The study adheres to STROBE guidelines for reporting observational research.

### Surgery

All patients were operated on using an anterolateral or posterolateral approach by either an orthopaedic resident (postgraduate year 5–6) or fellowship-trained joint reconstruction and trauma specialists. All implants used were either fully coated TAPERLOC^®^ implants (Zimmer Biomet, Warsaw, IN) or partially coated CORAIL^®^ implants (DePuy Synthes, Raynham, MA). According to our department’s protocol, full weight bearing as tolerated and range of motion exercises following HA are routinely initiated on postoperative day one. In cases of IPFF, However, the decision regarding weight-bearing status and range of motion was made intraoperatively by the surgeon based on fracture stability and adequacy of fixation.

### Outcome parameters

The primary outcomes included the rate of IPFF, the location of IPFF, and the effect of the IPFF on the postoperative physical therapy protocol. Secondary outcomes included operating time, 30-day, 90-day, and 1-year mortality, all-cause 90-day readmission to an orthopaedic department, revision rates, and indication for revision surgery.

### Statistical analysis

Statistical analysis was conducted using SPSS version 25 (IBM Corp., Armonk, NY). Continuous variables were presented as means with standard deviations (SD), while categorical variables were summarized as frequencies and percentages. Categorical variables were compared using the chi-square test. Normality of continuous variables was assessed with the Shapiro-Wilk test. For normally distributed variables, independent samples *t*-tests were used; for ordinal or non-normally distributed data, the Wilcoxon Mann-Whitney test was applied. To control for potential confounders and identify factors associated with IPFF, a multivariate logistic regression model was employed. All statistical tests were two-sided, with a significance threshold set at *P* < 0.05.

## Results

A total of 1,586 patients satisfied the final inclusion criteria. The case cohort consisted of 104 patients (6.6%) with IPFF. The control cohort consisted of 1,482 patients (93.4%) who did not have IPFF. No significant differences were noted for mean age (*p* = 0.701), mean BMI (*p* = 0.206), mean ASA score (*p* = 0.796), mean CCI (*p* = 0.878), and Dorr classification (*p* = 0.165) between the cohorts. The rate of female sex was significantly different between the groups (*p* = 0.038) (Table 1).

**Table 1:** Total cohort demographics.

	IPFF	non-IPFF	*p-*value
	(*n* = 104)	(*n* = 1482)	
Mean age, years (SD)	84.9 (6.7)	84.5 (6.4)	0.701
Female sex, *n* (%)	77 (74.0)	948 (64.0)	0.038
Mean BMI, kg/m^2^ (SD)	23.1 (3.6)	24.3 (4.2)	0.206
ASA score, *n* (%)			0.796
1	1 (0.9)	15 (1.0)	
2	33 (31.7)	534 (36.0)	
3	62 (59.6)	802 (54.1)	
4	8 (7.6)	112 (7.5)	
Missing	0 (0)	19 (1.2)	
Mean CCI, *n* (SD)	4.9 (1.5)	4.7 (1.5)	0.878
Surgical approach			0.478
Anterolateral	100 (96.1)	1401 (94.5)	
Posterolateral	4 (0.38)	81 (5.5)	
Dorr classification, *n* (%)			0.165
1	8 (7.6)	153 (10.3)	
2	49 (47.1)	521 (35.1)	
3	10 (9.6)	173 (11.6)	
Missing	37 (35.5)	635 (42.8)	
Follow-up, months (SD)	39.6 (26)	50.3 (28.1)	0.009

BMI, body mass index; ASA, American Society of Anaesthesiology; CCI, Charlson comorbidity index.

The distribution of IPFF locations from most common to least common was as follows: calcar (59.6%), greater trochanter (GT) (35.5%), and femoral shaft (8.6%). Most fractures were treated by fixation with wires or cables (92.3%), and postoperative weight-bearing status was mostly full weight-bearing (FWB) (95.1%) (Table 2).

**Table 2:** IPFF classification and treatment.

Implant	IPFF
	(*n* = 104)
Fracture location, *n* (%)	
Greater Trochanter	37 (35.5)
Calcar	62 (59.6)
Femur shaft	9 (8.6)
Fracture treatment, *n* (%)	
No treatment needed	2 (1.9)
Fixation with wires/cable	96 (92.3)
Stem revision	6 (5.7)
Post-surgery PT, *n* (%)	
FWB	99 (95.1)
PWB	3 (2.8)
NWB	2 (1.9)

IPFF, Intraoperative periprosthetic femoral fracture; PT, Physical therapy; FWB, Full weight bearing; PWB, Partial weight bearing; NWB, No weight bearing.

The mean surgery duration was significantly different between groups (97.2 min in the IPFF group vs. 73.6 min in the non-IPFF group, *p* < 0.001). No significant difference was found regarding the rates of mortality at 30 days (*p* = 0.087), 90 days (*p* = 0.151), and 1 year post-operatively (*p* = 0.531). All-cause 90-day readmission to an orthopedic department was comparable between groups (*p* = 0.306), as was the rate of revisions at the latest follow-up (*p* = 0.693). The most common indications for revision were PJI, periprosthetic fracture, and dislocation. No revisions were performed due to aseptic loosening in both groups. The distribution of the indications for these revisions (*p* = 0.681) was similar between the two groups (Table 3).

**Table 3:** Outcome parameters.

	IPFF	non-IPFF	*p-*value
	(*n* = 104)	(*n* = 1482)	
Mean surgery duration, min (SD)	97.2 (39.7)	73.6 (25.3)	< 0.001
Mortality, *n* (%)			
30-day	2 (1.9)	8 (5.9)	0.087
90-day	6 (5.8)	150 (10.1)	0.151
1-year	18 (17.3)	294 (19.8)	0.531
90-day readmission to orthopaedic department, *n* (%)	20 (19.2)	229 (15.5)	0.306
Revisions at latest follow-up, *n* (%)	5 (4.8)	85 (5.7)	0.693
Indication for revision, *n* (%)			0.681
Periprosthetic fracture	1 (0.9)	13 (0.8)	
PJI	3 (2.8)	53 (3.5)	
Aseptic loosening	0 (0)	0 (0)	
Dislocation	0 (0)	19 (1.2)	

IPFF, Intraoperative prosthetic femoral fracture; PJI, Periprosthetic joint infection.

A multivariate binary logistic regression has shown that IPFF was not associated with 30-day mortality (OR: 0.44; 95% CI: 0.10–1.90; *p* = 0.274), 90-day mortality (OR: 0.58; 95% CI: 0.22–1.51; *p* = 0.270), or 1-year mortality (OR: 0.88; 95% CI: 0.48–1.60; *p* = 0.682) (Table 4).

**Table 4:** Logistic regression model for mortality.

Variable	30-day mortality		90-day mortality		1-year mortality	
	*p*-value	OR (95% CI)	*p*-value	OR (95% CI)	*p*-value	OR (95% CI)
IPFF	0.274	0.44 (0.10–1.90)	0.270	0.58 (0.22–1.51)	0.682	0.88 (0.48–1.60)
Male gender	0.168	1.50 (0.84–2.70)	0.008	1.77 (1.16–2.71)	<0.001	1.76 (1.29–2.40)
Age	0.105	1.03 (0.99–1.08)	0.016	1.04 (1.00–1.07)	<0.001	1.04 (1.01–1.07)
ASA score	<0.001	3.72 (2.25–6.15)	<0.001	1.98 (1.39–2.84)	<0.001	1.93 (1.49–2.52)
BMI	0.706	1.01 (0.94–1.08)	0.091	0.95 (0.90–1.00)	0.021	0.95 (0.91–0.99)
CCI	0.047	1.17 (1.00–1.37)	0.234	1.07 (0.95–1.22)	0.192	1.06 (0.96–1.17)

IPFF, Intraoperative prosthetic femoral fracture; ASA, American Society of Anaesthesiology; BMI, body mass index; CCI, Charlson comorbidity index.

## Discussion

The main findings of this study are that, despite longer operative times, patients with IPFF had similar rates of mortality, readmission, and revision compared to those without IPFF, suggesting no adverse impact on long-term outcomes when managed appropriately.

IPFF is a well-documented complication following hip arthroplasty [11, 14, 19, 25–27]. While the majority of studies have investigated this complication in elective THA for patients who underwent preoperative optimization, only several studies specifically focused on patients who underwent cementless HA for displaced FNF [16, 23, 28–30]. In these cases, the patients are generally less healthy, have more comorbidities, and undergo urgent surgery with no preoperative optimization. [16, 28, 30, 31].

Our cohort found an incidence of 6.6% of patients with IPFF, consistent with previous rates reported for cementless HA following displaced FNF: DeAngelis et al. reported rates of 4.7% [29], Taylor et al. observed an incidence of 7.5% [28], Mazzawi et al. reported the incidence of 12.2% [21], Unnanuntana and Saiyudthong reported an incidence of 17.7% [22], and Hong et al. reported an incidence of 14.7%, compared to 5.4% for cemented HA [16].

The location and treatment of IPFF in our study are similar to previous studies, where calcar and GT fractures were the most common, and most of the fractures were treated with ORIF using cerclage wiring, while a minority were managed with stem revision [31, 32].

Following HA for displaced FNF, Unnanuntana and Saiyudthong found worse short-term outcomes in the IPFF group, as measured by longer operative time, extended hospital stays, and higher blood loss compared to the non-IPFF group. However, for long-term outcomes, after a 3-year follow-up, there were no significant differences in VAS scores, stem position, or loosening between the groups [22] Similarly, Lee et al. in his study for similar patients, Reported that while the IPFF group had worse short-term outcomes, such as non-weight bearing status and more wheelchair-bound patients, though their 1-year long-term outcomes were not compromised [23]. These results are consistent with findings for IPFF following cementless elective THA. In a study by Berend et al. [35], with a 7.5-year follow-up, no revisions were needed, and mid- to long-term outcomes remained positive in the IPFF group following elective THA.

It is believed that adhering to principles that ensure the stability of the implant, prevent fracture propagation, and maintain proper alignment at the end of the surgery, offers the best chance for good long-term outcomes [27, 33]. Thillemann et al. [11] reported that the risk of revision surgery after IPFF is highest in the first 6 months, but if the femoral stem is stable and the fracture heals, long-term outcomes are not affected. In our study, most patients in the IPFF group were allowed to fully weight-bearing immediately postoperatively, which could indicate that stability of the implant was restored intraoperatively, and therefore long-term outcomes were not affected. This approach aligns with the literature, which suggests that if the fracture pattern and implant are deemed stable, patients may be allowed protected or full weight bearing with close clinical and radiographic follow-up [6].

This study aims to review the incidence of IPFF and compare long-term postoperative outcomes between patients who sustained an IPFF during cementless HA for displaced FNF and those who did not. Our findings show no significant long-term difference between the groups. To our knowledge, this is the largest study to date on this topic, with the most extended follow-up. The median follow-up was approximately 50 months, though longer-term follow-up is challenging due to the limited life expectancy of this patient population. Notably, 19.8% and 17.3% patients in the IPPF and the non-IPFF groups, respectively, died within one year, emphasizing the high mortality rate in this population. This is comparable to the rates reported in previous studies [28, 29], where pre-existing conditions may be the cause of the high mortality rate.

This study has several limitations. Its retrospective design may introduce selection bias and loss-to-follow-up bias; differences or missing data in baseline patient characteristics could have influenced the results. We acknowledge the possibility that some cases of IPFF may have gone undiagnosed despite our thorough intraoperative assessment and postoperative follow-up protocols. However, we believe this is minimal because the majority of IPFF cases are documented in the operative and medical records. Most importantly, we did not collect or assess functional outcomes such as gait performance or pain scores. This limits our ability to evaluate the full clinical impact of IPFF beyond survival and revision rates. Additionally, Trendelenburg gait, a common sequela following GT fractures [34], were not assessed, further limiting functional interpretation.

## Conclusion

While IPFF remains a recognized complication of cementless HA for displaced FNF, when properly managed intraoperatively, including stable fixation to support implant stability, it does not negatively impact long-term outcomes.

## Data Availability

Data are not available for this study.
